# Supplementary material for: A self-improving triboelectric nanogenerator with improved charge density and increased charge accumulation speed
Source: Nat Commun. 2018 Sep 14;9:3773. doi: 10.1038/s41467-018-06045-z (PMC6138706; doi:10.1038/s41467-018-06045-z)
Supplement: Supplementary file 1 — Supplementary Information [file 41467_2018_6045_MOESM1_ESM.pdf]

Supplementary Information

A Self-improving Triboelectric Nanogenerator with Improved Charge Density and Increased Charge Accumulation Speed

Li Cheng[1], Qi Xu[2], Youbin Zheng[3], Xiaofeng Jia[2], Yong Qin[2,*]

[1]Zhongyuan University of Technology, Zhengzhou, Henan 450007, China.

[2]Institute of Nanoscience and Nanotechnology, Lanzhou University, Gansu 730000, China.

[3]State Key Laboratory of Solid Lubrication, Lanzhou Institute of Chemical Physics, Chinese Academy of Sciences, Lanzhou 730000, China.

Correspondence and requests for materials should be addressed to Y.Q. (email: qinyong@lzu.edu.cn)

Supplementary Note 1.

Choice of materials. Materials used in the SI-TENG are chosen for the following reason. PVDF and PA-6 are chosen as friction layers of the part I, because PVDF and PA-6 are respectively high negative and positive in the triboelectric series, and they are easy to generate negative and positive charge in the friction process. PVDF/EP films are chosen as the insulating films, because dielectric coefficient of PVDF are relatively high in polymers, thus, the capacitance between electrodes 1 and 2 is higher, and the charge density filled into the PPCS could be higher. Because the spin-coated PVDF film is not compact, EP was chosen to make the film compact to ignore the insulating film breakdown under high voltage.

Supplementary Note 2.

Theoretical study of SI-TENG's working process.

The equivalent circuit of the SI-TENG is shown in Supplementary Fig. 8, where, C_1 represents the capacitance between two friction layers, C_2 represents the capacitance between one friction layer and the corresponding electrode in part I, C_3 represents the capacitance between electrodes 1 and 2 in part II, C_4 represents the capacitance between electrodes 1 and 3 or electrodes 2 and 4 in part II. σ_1, σ_2, σ_3 and σ_4 represent the equivalent charge densities in the equivalent capacitors, and the sum of σ_1 and σ_2 equals to the charge density of part I (marked as σ_0), the sum of σ_3 and σ_4 equals to the charge density filled into the PPCS (marked as σ), the variation of σ_4 equals to the effective charge density of the SI-TENG (marked as $\Delta\sigma$).

The voltage and the capacitance are determined by the following equations,

$$V = \frac{\sigma S}{C} \quad (1)$$

$$C = a\frac{\varepsilon\varepsilon_0 S}{d} \quad (2)$$

here, S is the area of the electrodes (equal for C_1, C_2, C_3 and C_4), a is a dimensionless shape factor to correct the shape of the capacitance deviated to a plane-parallel capacitor (if the gap thickness of the capacitance is much smaller than its length, a = 1), ε is the permittivity of the gap materials in the capacitances, ε_0 is the

permittivity of vacuum and d is the gap thickness of the capacitances (d_1, d_2, d_3 and d_4 respectively for C_1, C_2, C_3 and C_4).

When the SI-TENG reaches the stable state, the output voltage of part I should be equal to the voltage of C_3 or the sum of voltage of two C_4. Thus,

$$\left|\frac{\sigma_1 S}{C_1} - 2\frac{\sigma_2 S}{C_2}\right| = V = \frac{\sigma_3 S}{C_3} = 2\frac{\sigma_4 S}{C_4} \quad (3)$$

$$\left|\frac{\sigma_1 S}{C_1} - 2\frac{\sigma_2 S}{C_2}\right| = \frac{\sigma S}{C_3 + \frac{C_4}{2}} \quad (4)$$

In these equations, after the SI-TENG reaches the stable state, σ_1, σ_2 and σ remain stable (because of the existence of the rectifier bridge), and C_1 and C_3 change with the working of the device, at the pressed and released states, equation 4 could be divided to the following two equations (the subscripts p and r respectively indicate the pressed and released state),

$$\frac{\sigma_1 S}{C_{1p}} - 2\frac{\sigma_2 S}{C_2} = -\frac{\sigma S}{C_{3p} + \frac{C_4}{2}} \quad (5)$$

$$\frac{\sigma_1 S}{C_{1r}} - 2\frac{\sigma_2 S}{C_2} = \frac{\sigma S}{C_{3r} + \frac{C_4}{2}} \quad (6)$$

From equations 5 and 6, we can obtain the relationship between σ_1 and σ_2,

$$\frac{\sigma_2}{\sigma_1} = \frac{\left(\frac{2C_{3p}+C_4}{2C_{1p}} + \frac{2C_{3r}+C_4}{2C_{1r}}\right)}{2\frac{C_{3p}+C_{3r}+C_4}{C_2}} \quad (7)$$

Because, $d_{1p} \approx 0$, $d_{3r} \gg d_4$, $d_{3r} \gg d_{3p}$, $d_{1r} \gg d_2$, $d_{1r} \gg d_4$, we can get $1/C_{1p} \approx 0$, $C_{3r} \ll C_4$, $C_{3r} \ll C_{3p}$, $C_{1r} \ll C_2$, $C_{1r} \ll C_4$, equation 7 could be simplified as

$$\frac{\sigma_2}{\sigma_1} \approx \frac{C_4 C_2}{4(C_{3p}+C_4)C_{1r}} \quad (8)$$

Thus, we can get $\sigma_2 \gg \sigma_1$, and $\sigma_2 \approx \sigma_0$. From equation 5, we can get

$$\sigma \approx \frac{\sigma_0(2C_{3p}+C_4)}{C_2} \quad (9)$$

The relationship between $\Delta\sigma$ and σ could be calculated by following way. From equation 3, the relationship between σ_4 and σ is

$$\sigma_4 = \frac{C_4}{C_4+2C_3}\sigma \quad (10)$$

At the pressed and released states, this equation could be divided to the following two equations,

$$\sigma_{4p} = \frac{C_4}{C_4+2C_{3p}}\sigma \tag{11}$$

$$\sigma_{4r} = \frac{C_4}{C_4+2C_{3r}}\sigma \tag{12}$$

The effective charge density of the SI-TENG is

$$\Delta\sigma = \left(\frac{C_4}{C_4+2C_{3r}} - \frac{C_4}{C_4+2C_{3p}}\right)\sigma \approx \frac{2C_{3p}}{C_4+2C_{3p}}\sigma \tag{13}$$

From equations 2, 3, 9 and 13, the relationship of the effective charge density of SI-TENG with part I's charge density or output voltage are

$$\Delta\sigma \approx \frac{2C_{3p}}{C_2}\sigma_0 = \frac{2\varepsilon_3 d_2}{\varepsilon_2 d_{3p}}\sigma_0 \tag{14}$$

$$\Delta\sigma \approx \frac{\varepsilon_3\varepsilon_0}{d_{3p}}V \tag{15}$$

If we set the permittivity of the insulating films in the PPCS as ε ($\varepsilon=\varepsilon_3$), and thickness of the insulating films in the PPCS as d ($d=d_3/2$), equations 14 and 15 are

$$\Delta\sigma \approx \frac{\varepsilon d_2}{\varepsilon_2 d}\sigma_0 \tag{16}$$

$$\Delta\sigma \approx \frac{\varepsilon\varepsilon_0}{2d}V \tag{17}$$

Supplementary Table 1|Representative result of TENGs with high charge density.

Materials	Driving mode	Special treatment	Charge density	Ref.
**PVC NWs & Al**	Sliding	ICP treatment	115 μC m^{-2}	1
**Silicone ecoflex**	Finger press	Oxygen plasma treatment	140 μC m^{-2}	2
**PVDF & Nylon**	Contact-separate	Prior-charge injection	148 μC m^{-2}	3
**PVDF NWs & Nylon NWs**	Contact-separate	Fabricated by electrospun	190 μC m^{-2}	4
**PA**	Contact-separate	Sliding one side with FEP in advance	211 μC m^{-2}	5
**FEP & Al**	Contact-separate	Ionized air injection	240 μC m^{-2}	6
**PTFE & Cu**	Contact-separate	In vacuum	1003 μC m^{-2}	7

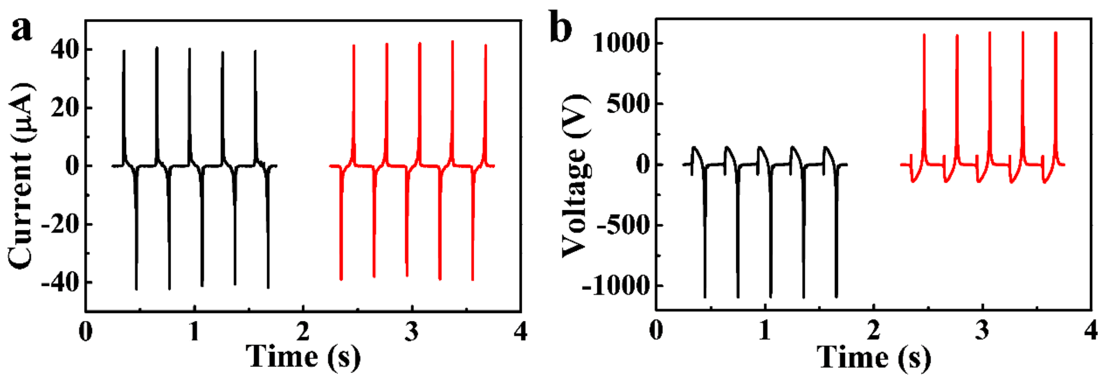

Supplementary Figure 1|Output of the SI-TENG after reversing the connection of the rectifier bridge and the PPCS. (**a**) and (**b**) respectively show the output current and voltage of the SI-TENG before (black) and after (red) reverse connecting.

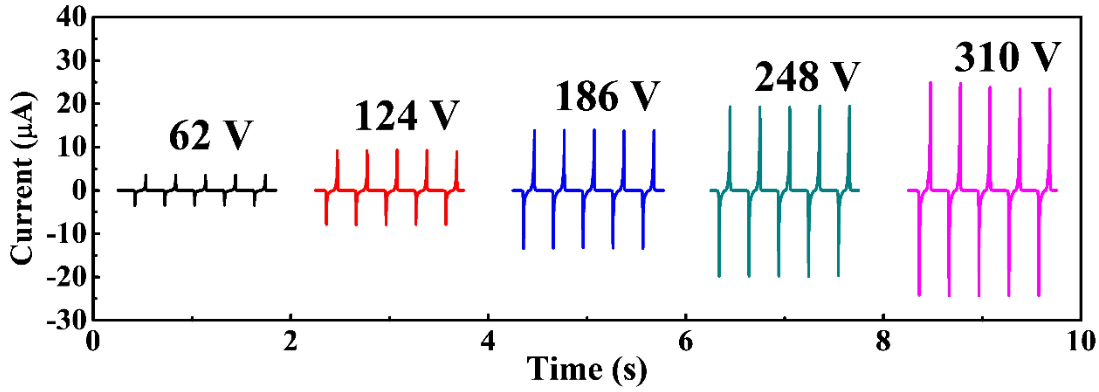

Supplementary Figure 2|Output currents of the SI-TENG with part I's voltage changes from 62 V to 310 V. The voltages of the part I are marked above the curves.

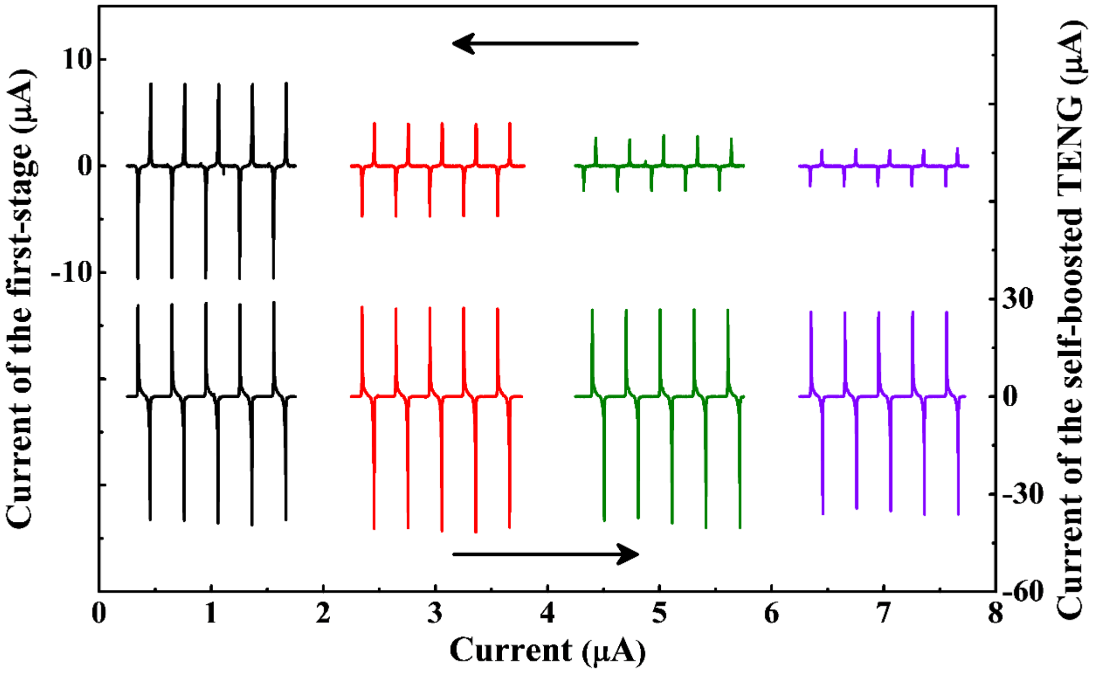

Supplementary Figure 3|Output currents of the SI-TENG with different output current of the part I. The curves on the top of the image show the output current of the part I, and the curves on the bottom show the corresponded output current of the SI-TENG.

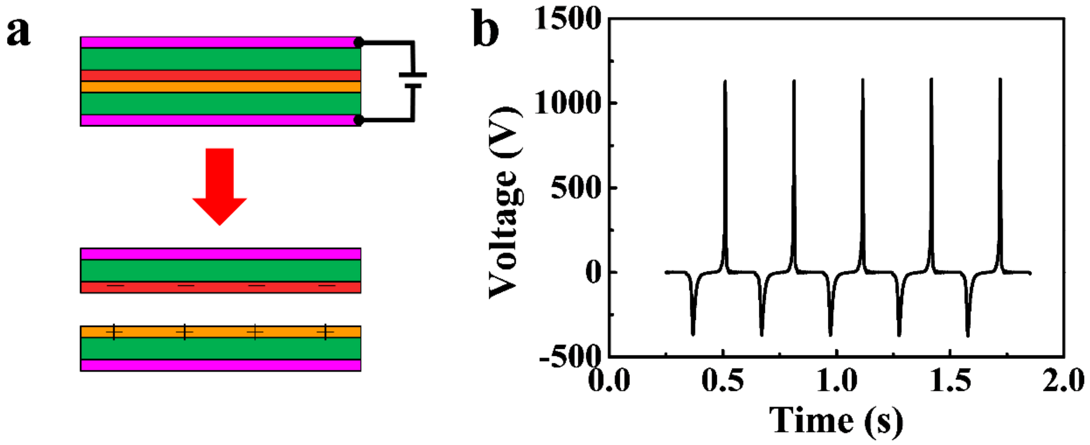

Supplementary Figure 4|Schematic image of the charge injection process of the part I (a), and its output voltage after charge injection (**b**).

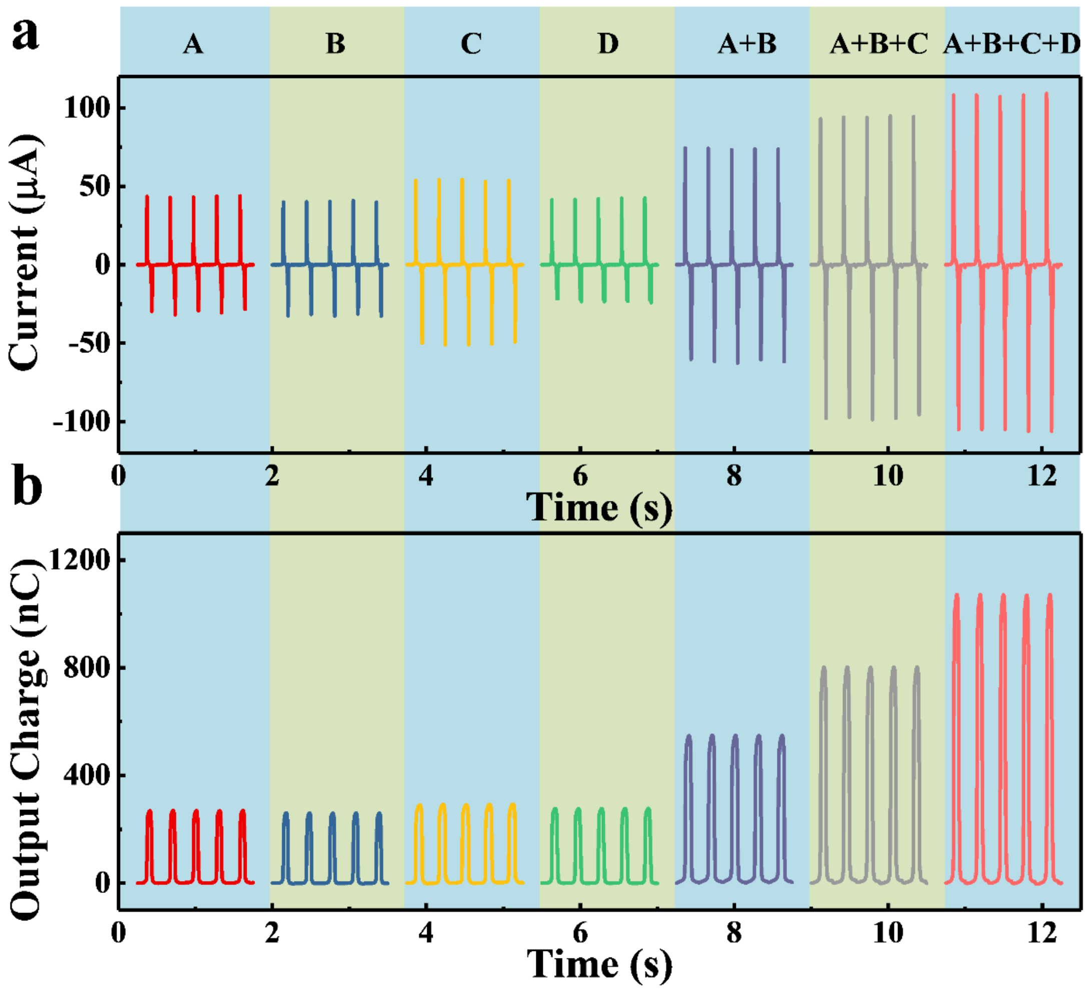

Supplementary Figure 5|Output of the multilayer SI-TENG. (**a**) and (**b**) respectively shows output current and charge generated by four SI-TENG (using different part II devices, marked as A B C and D) and the multilayer SI-TENGs comprised of two (A and B), three (A, B and C) or four (A, B, C and D) part IIs.

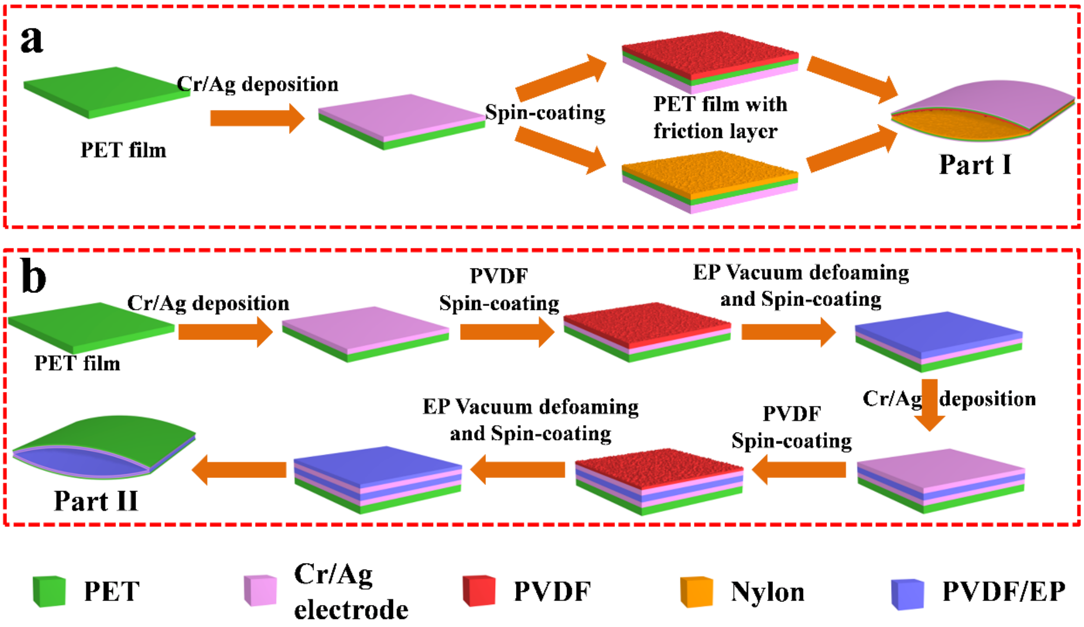

Supplementary Figure 6|Fabrication process of the SI-TENG. (**a**) and (**b**) respectively shows the fabrication process of the part I and the part II.

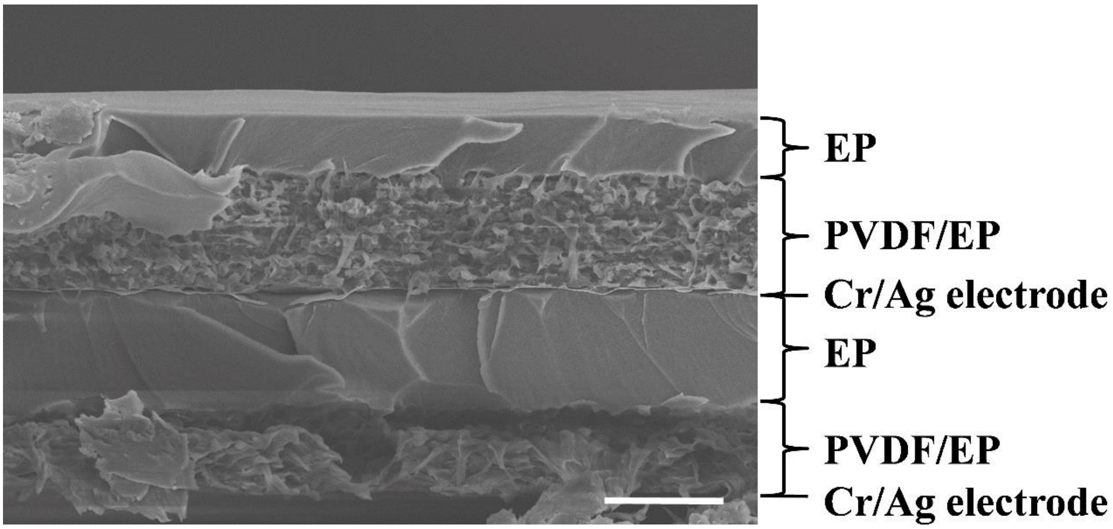

Supplementary Figure 7|Cross-sectional view SEM image of structure fabricated on the part II. The scale bar is 10 μm.

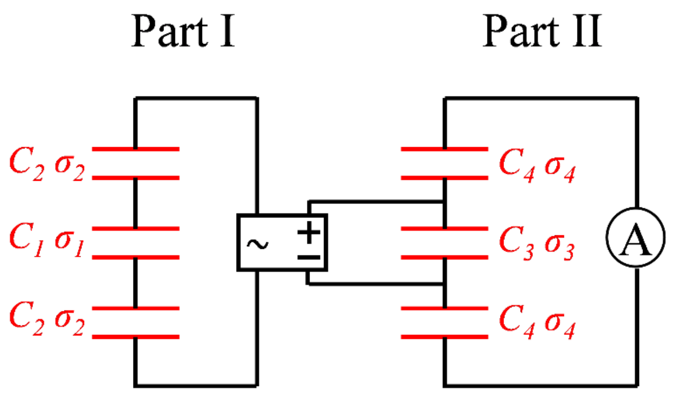

Supplementary Figure 8|Equivalent circuit of the SI-TENG.

Supplementary References

1. Du, W., et al. A Three Dimensional Multi-Layered Sliding Triboelectric Nanogenerator. *Adv. Energy Mater.* **4**, 1301592 (2014).
2. Li, S., et al. All-Elastomer-Based Triboelectric Nanogenerator as a Keyboard Cover To Harvest Typing Energy. *ACS Nano* **10**, 7973-7981 (2016).
3. Wang, Z., Cheng, L., Zheng, Y., Qin, Y. & Wang, Z. L. Enhancing the performance of triboelectric nanogenerator through prior-charge injection and its application on self-powered anticorrosion. *Nano Energy* **10**, 37-43 (2014).
4. Zheng, Y. *et al.* An electrospun nanowire-based triboelectric nanogenerator and its application in a fully self-powered UV detector. *Nanoscale* **6**, 7842-7846 (2014).
5. Wei, X.Y., Zhu, G., & Wang, Z. L. Surface-charge engineering for high-performance triboelectric nanogenerator based on identical electrification materials. *Nano Energy* **10**, 83-89 (2014).
6. Wang, S. *et al.* Maximum Surface Charge Density for Triboelectric Nanogenerators Achieved by Ionized-Air Injection: Methodology and Theoretical Understanding. *Adv. Mater.* **26**, 6720-6728 (2014).
7. Wang, J. *et al.* Achieving ultrahigh triboelectric charge density for efficient energy harvesting. *Nat. Commun.* **8**, 88 (2017).
