# Supplementary material for: A self-improving triboelectric nanogenerator with improved charge density and increased charge accumulation speed
Source: Nat Commun. 2018 Sep 14;9:3773. doi: 10.1038/s41467-018-06045-z (PMC6138706; doi:10.1038/s41467-018-06045-z)
Supplement: Supplementary file 3 — Description of Additional Supplementary Files [file 41467_2018_6045_MOESM3_ESM.pdf]

Descriptions of Additional Supplementary Files:

File Name: Supplementary Movie 1
Description: The video shows the SI-TENG's output voltage changes with the process of charge filled in the PPCS.

File Name: Supplementary Movie 2
Description: The video shows the SI-TENG's output current changes with the process of charge filled in the PPCS.
